# Comparison of NMR and crystal structures highlights conformational isomerism in protein active sites

**DOI:** 10.1107/S1744309110033658

**Published:** 2010-09-30

**Authors:** Pedro Serrano, Bill Pedrini, Michael Geralt, Kristaps Jaudzems, Biswaranjan Mohanty, Reto Horst, Torsten Herrmann, Marc-André Elsliger, Ian A. Wilson, Kurt Wüthrich

**Affiliations:** a Department of Molecular Biology, The Scripps Research Institute, La Jolla, CA 92037, USA; b Joint Center for Structural Genomics, http://www.jcsg.org, USA; c Institute of Molecular Biology and Biophysics, ETH Zürich, CH-8093 Zürich, Switzerland; d Centre Européen de RMN à Très Hauts Champs, Université de Lyon FRE 3008 CNRS, F-69100 Villeurbanne, France; e Skaggs Institute of Chemical Biology, The Scripps Research Institute, La Jolla, CA 92037, USA

**Keywords:** *Thermotoga maritima* anti-σ factor antagonist, mouse γ-glutamylamine cyclotransferase, NMR and crystal structure comparison, active-site conformation

## Abstract

The JCSG has recently developed a protocol for systematic comparisons of high-quality crystal and NMR structures of proteins. In this paper, the extent to which this approach can provide function-related information on the two functionally annotated proteins TM1081, a *Thermotoga maritima* anti-σ factor antagonist, and A2LD1 (gi:13879369), a mouse γ-glutamylamine cyclotransferase, is explored. The NMR structures of the two proteins have been determined in solution at 313 and 298 K, respectively, using the current JCSG protocol based on the software package *UNIO* for extensive automation. The corresponding crystal structures were solved by the JCSG at 100 K and 1.6 Å resolution and at 100 K and 1.9 Å resolution, respectively. The NMR and crystal structures of the two proteins share the same overall molecular architectures. However, the precision of the structure determination along the amino-acid sequence varies over a significantly wider range in the NMR structures than in the crystal structures. Thereby, in each of the two NMR structures about 65% of the residues have displacements below the average and in both proteins the less well ordered residues include large parts of the active sites, in addition to some highly solvent-exposed surface areas. Whereas the latter show increased disorder in the crystal and in solution, the active-site regions display increased displacements only in the NMR structures, where they undergo local conformational exchange on the millisecond time scale that appears to be frozen in the crystals. These observations suggest that a search for molecular regions showing increased structural disorder and slow dynamic processes in solution while being well ordered in the corresponding crystal structure might be a valid initial step in the challenge of identifying putative active sites in functionally unannotated proteins with known three-dimensional structure.

## Abstract 2

Tools for systematic comparisons of NMR and crystal structures developed by the JCSG were applied to two proteins with known functions: the *T. maritima* anti-σ factor antagonist TM1081 and the mouse γ-glutamylamine cyclotransferase A2LD1 (gi:13879369). In an attempt to exploit the complementarity of crystal and NMR data, the combined use of the two structure-determination techniques was explored for the initial steps in the challenge of searching proteins of unknown functions for putative active sites.

## 1. Introduction

A recently introduced JCSG protocol for systematic comparisons of NMR and crystal structures (Jaudzems *et al.*, 2010 ▶; Mohanty *et al.*, 2010 ▶) is used with two functionally annotated proteins: the anti-σ factor antagonist TM1081 from *Thermotoga maritima* and the *Mus musculus* γ-glutamylamine cyclotransferase A2LD1 (GGACT; gi:13879369). In an attempt to exploit the complementarity of NMR spectroscopy and X-ray crystallography in providing function-related information, we explore the combined use of the two structure-determination techniques for initial identification of putative active sites in proteins of unknown function.

TM1081 is annotated as an anti-σ factor antagonist based on sequence similarity to members of the STAS (sulfate transporter and anti-σ factor antagonist) Pfam family (PF01740). This domain, which is often found in the C-terminal region of sulfate transporters and bacterial anti-σ factor antagonists, may have a general NTP-binding function (Aravind & Koonin, 2000 ▶). TM1081 shares more than 30% sequence identity with its Thermotogae, Spirochaetes and Actinobacteria counterparts, which possess the anticipated anti-σ factor antagonist fold (Seavers *et al.*, 2001 ▶; Masuda *et al.*, 2004 ▶; Lee *et al.*, 2004 ▶), indicating that the *Thermotoga* protein may also be involved in transcriptional regulation of gene expression as part of cell-adaptation mechanisms that are mediated by a variety of stress-response signals. The TM1081 crystal structure has been determined by the JCSG (PDB entry 3f43).

When the crystal structure of the mouse protein A2LD1 (gi:13879369) was determined by the JCSG (PDB entry 1vkb), it was a ‘domain of unknown function’ and classified as a new fold (Klock *et al.*, 2005 ▶). This protein belongs to the highly conserved Pfam AIG2 family (PF03674), which includes hundreds of members from all kingdoms of life, and was subsequently annotated as an AIG2-like domain-containing protein-1. Recently, human γ-glutamylamine cyclotransferase (GCACT) was structurally (PDB code 3jud) and biochemically characterized based on homology with the JCSG mouse homolog structure (Oakley *et al.*, 2010 ▶). The proteins share 72% sequence identity and adopt very similar structures, including a conserved catalytic site, strongly indicating that the mouse protein is also a γ-glutamylamine cyclotransferase.

Here, we describe NMR structure determinations of TM1081 and A2LD1 using the current JCSG protocol, which makes use of the *UNIO* software package for extensive automation (Herrmann *et al.*, 2002*a* ▶,*b* ▶; Volk *et al.*, 2008 ▶; Fiorito *et al.*, 2008 ▶). For comparison of the newly determined NMR structures with the aforementioned crystal structures, we continue to explore the recently introduced strategy of using ‘reference crystal structures’ (RefCrystal) and ‘reference NMR structures’ (RefNMR) (Jaudzems *et al.*, 2010 ▶) to analyze and support the identification of structure variations that arise from the different environments in the crystal and in solution rather than from the different structure-determination techniques.

## 2. Methods and experiments

### 2.1. Preparation of TM1081

The vector MH4a containing the TM1081 gene with an N-terminal expression and polyhistidine purification tag was cloned by the JCSG Crystallomics Core and used to produce the proteins for both the NMR and crystal structure determinations. For NMR studies, ^15^N,^13^C-labeled TM1081 was expressed using *Escherichia coli* strain Rosetta (DE3) (Novagen) and M9 minimal media containing either 1 g l^−1^ ^15^NH_4_Cl and 4 g l^−1^ unlabeled d-glucose or 1 g l^−1^ ^15^NH_4_Cl and 4 g l^−1^ [^13^C_6_]-d-glucose (Cambridge Isotope Laboratories) as the sole sources of nitrogen and carbon. After the addition of 100 mg l^−1^ ampicillin and 20 mg l^−1^ chloramphenicol, the cells were grown at 310 K to an OD_600_ of 0.64, induced with 1 m*M* isopropyl β-d-1-thiogalactopyranoside (IPTG) and grown for a further 3.5 h to a final OD_600_ of 1.15. The cells were harvested by centrifugation at 5000*g* for 5 min at 277 K and frozen at 253 K overnight. The next day, the cell pellet was thawed and resuspended in 53 ml buffer *A* (20 m*M* sodium phosphate pH 7.4, 300 m*M* NaCl, 30 m*M* imidazole) containing one Complete EDTA-free protease-inhibitor cocktail tablet (Roche) and lysed by ultrasonication. The soluble fraction of the cell lysate was isolated by centrifugation at 20 000*g* for 30 min at 277 K, decanted and filtered through a 0.22 µm filter. The solution was then incubated in a 348 K water bath for 30 min. The precipitated material was removed by centrifugation at 8000*g* for 30 min at 277 K. The supernatant was recovered and passed through the 0.22 µm filter before application onto a 5 ml HisTrap HP column (GE Healthcare) pre-equilibrated in buffer *A*. The bound protein was eluted using a linear 30–500 m*M* imidazole gradient over a 100 ml volume. Fractions containing the protein were pooled and applied onto a HiLoad 26/60 column of Superdex 75 gel-filtration resin (GE Healthcare) pre-equilibrated in NMR buffer (20 m*M* sodium phosphate pH 5.7, 150 m*M* NaCl). The fractions containing TM1081 were pooled and concentrated from 24 ml to 500 µl by ultrafiltration using an Amicon ultracentrifugal filter device with 5 kDa molecular-weight cutoff (Millipore). All purification steps were monitored by SDS–PAGE. The yield of purified TM1081 was 14.9 mg per litre of culture.

NMR samples were prepared by adding 5%(*v*/*v*) D_2_O and 0.03%(*w*/*v*) NaN_3_ to 500 µl of a 1.0 m*M* solution of ^15^N,^13^C-labeled TM1081 in NMR buffer.

### 2.2. Preparation of A2LD1

The plasmid vector MH4a-A2LD1 (gi:13879369) obtained from the JCSG Crystallomics Core was used as the template for PCR amplification with the primers 5′-CCG**CAT*ATG***GCCCACATCTTCGTGTATGGCA-3′ and 5′-CGG**AAGCTT** *CTA*TTATCTGTTTTCCCGGGGGTTGTAGCG-3′, where the *Nde*I and *Hin*dIII restriction sites are shown in bold and the initiation and stop codons are italicized. The PCR product was digested with *Nde*I and *Hin*dIII and inserted into the same restriction sites of the pET-25b vector after treatment with calf intestinal alkaline phosphatase (CIP). The resulting plasmid pET-25b-gi:13879369 was used to transform *E. coli* strain Rosetta (DE3) (Novagen) and the protein was expressed in M9 minimal media containing either 1 g l^−1^ ^15^NH_4_Cl and 4 g l^−1^ un­labeled d-glucose or 1 g l^−1^ ^15^NH_4_Cl and 4 g l^−1^ [^13^C_6_]-d-glucose (Cambridge Isotope Laboratories) as the sole sources of nitrogen and carbon. After the addition of 100 mg l^−1^ ampicillin, the cells were grown at 310 K to an OD_600_ of 0.44, induced with 1 m*M* isopropyl β-­d-1-thiogalactopyranoside (IPTG) and grown for a further 3 h to a final OD_600_ of 0.87. The cells were harvested by centrifugation at 5000*g* for 5 min at 277 K and frozen at 253 K overnight. The next day, the cell pellet was thawed and resuspended in 38 ml buffer *B* (25 m*M* sodium phosphate at pH 7.6, 25 m*M* NaCl, 2 m*M* DTT) containing one Complete protease-inhibitor cocktail tablet (Roche) and lysed by ultrasonication. The soluble fraction of the cell lysate was isolated by centrifugation at 20 000*g* for 30 min at 277 K, decanted and filtered through a 0.22 µm filter. The solution was then applied onto a 5 ml HiTrap QHP column (GE Healthcare) pre-equilibrated in buffer *B*. Initially, A2LD1 eluted in the second half of the flowthrough during sample injection. The flowthrough fractions containing A2LD1 were pooled and again applied onto a 5 ml HiTrap QHP column pre-equilibrated in buffer *B*; the protein bound and was subsequently eluted from the column with 125 m*M* NaCl. Fractions containing the protein were concentrated to 10 ml by ultrafiltration using an Amicon ultracentrifugal filter device with 5 kDa molecular-weight cutoff (Millipore) and were then applied onto a HiLoad 26/60 column of Superdex 75 gel-filtration resin (GE Healthcare) pre-equilibrated in NMR buffer (25 m*M* sodium phosphate pH 6.8, 50 m*M* NaCl, 0.5 m*M* DTT). The fractions containing A2LD1 were pooled and concentrated from 60 ml to 500 µl by ultrafiltration. All purification steps were monitored by SDS–PAGE. The yield of purified A2LD1 was 32.7 mg per litre of culture.

NMR samples were prepared by adding 10%(*v*/*v*) D_2_O, 4.5 m*M d*-­DTT and 0.03%(*w*/*v*) NaN_3_ to 500 µl of a 1.1 m*M* solution of ^15^N,^13^C-labeled A2DL1 in NMR buffer.

### 2.3. NMR spectroscopy

NMR experiments for the protein TM1081 were conducted at 313 K on Bruker Avance 600 and Avance 800 spectrometers equipped with TXI HCN *z*-gradient or *xyz*-gradient probes and the measurements for A2DL1 were performed at 298 K using the same spectrometers. Internal 2,2-dimethyl-2-silapentane-5-sulfonate (DSS) was used as a chemical shift reference (Wishart & Sykes, 1994 ▶). For the backbone resonance assignments of TM1081, we used a 2D [^15^N,^1^H]-HSQC spectrum (Mori *et al.*, 1996 ▶) and triple-resonance 3D HNCA, 3D HNCO, 3D HNCACB and 3D CBCA(CO)NH spectra (Bax & Grzesiek, 1993 ▶). For the side-chain assignments and the collection of conformational constraints, three NOESY spectra were recorded with a mixing time of 60 ms: 3D [^1^H,^1^H]-NOESY-^15^N-HSQC, 3D [^1^H,^1^H]-NOESY-^13^C(ali)-HSQC and 3D [^1^H,^1^H]-NOESY-^13^C(aro)-HSQC. The ^13^C carrier frequencies were at 27 and 125 p.p.m., respectively, for coverage of the aliphatic and aromatic spectral regions. For A2DL1, the backbone resonance assignments were based on three 600 MHz APSY-NMR data sets, *i.e.* 4D APSY-HACANH (38 projections), 5D APSY-HACACONH (22 projections) and 5D APSY-CBCACONH (24 projections) (Hiller *et al.*, 2008 ▶), and on a low-resolution 3D HNCA spectrum (Bax & Grzesiek, 1993 ▶). Side-chain assignments and the collection of conformational constraints were achieved using the same types of spectra and following the same procedure as for TM1081. In addition, a 2D [^15^N,^1^H]-HSQC spectrum (Mori *et al.*, 1996 ▶) and a heteronuclear 2D [^1^H]-NOE TROSY experiment (Zhu *et al.*, 2000 ▶) were recorded at 700 MHz on a Bruker DRX spectrometer.

### 2.4. NMR structure determination

For TM1081, sequence-specific backbone resonance assignments were obtained with the program *CARA* (Keller, 2004 ▶) from the aforementioned triple-resonance experiments. In a second interactive step, the assignments were extended to the α- and β-protons using the 3D [^1^H,^1^H]-NOESY-^15^N-HSQC and 3D [^1^H,^1^H]-NOESY-^13^C(ali)-HSQC data sets. Automated analysis of the three standard 3D heteronuclear-resolved [^1^H,^1^H]-NOESY data sets with the software *UNIO-ATNOS*/*ASCAN* (Herrmann *et al.*, 2002*a* ▶; Fiorito *et al.*, 2008 ▶) was then used to obtain amino-acid side-chain assignments.

For A2LD1, the NMR assignments were obtained as described for TM1081, except that the backbone assignments were extensively automated, using the three APSY-NMR spectra mentioned in the preceding sections as input for the software *UNIO-MATCH* (Volk *et al.*, 2008 ▶) and then validated interactively using the information contained in a low-resolution 3D HNCA spectrum.

For both proteins, automated structure calculation was performed using the software *UNIO-ATNOS*/*CANDID* (Herrmann *et al.*, 2002*a* ▶,*b* ▶) in combination with the torsion-angle dynamics program *CYANA* v.3.0 (Güntert *et al.*, 1997 ▶). The standard seven-cycle *UNIO-ATNOS/CANDID* protocol (Herrmann *et al.*, 2002*a* ▶) was employed with 80 randomized starting conformers. The 40 conformers with the lowest residual *CYANA* target-function values after cycle 7 were energy-minimized in a water shell with the program *OPALp* (Luginbühl *et al.*, 1996 ▶; Koradi *et al.*, 2000 ▶) using the AMBER force field (Cornell *et al.*, 1995 ▶). The 20 conformers with the lowest target-function values that satisfied the validation criteria (see below) were selected to represent the NMR structures and were analyzed using the program *MOLMOL* (Koradi *et al.*, 1996 ▶).

### 2.5. Structure validation and data deposition

Structure validation was performed as described in Jaudzems *et al.* (2010 ▶). The chemical shifts were deposited in the BioMagRes Bank (http://www.bmrb.wisc.edu; entry Nos. 10868 and 16380 for TM1081 and A2DL1, respectively) and the atomic coordinates of the bundles of 20 conformers used to represent the solution structures of TM1081 and A2DL1 have been deposited in the Protein Data Bank (http://www.rcsb.org/pdb/) with accession codes 2ka5 and 2kl2, respectively.

### 2.6. Calculation of reference crystal structures and reference NMR structures

Reference crystal structures and reference NMR structures were computed following the strategy introduced in Jaudzems *et al.* (2010 ▶). For the reference crystal structure, the positions of the H atoms in the crystal were calculated using the standard residue geometries from the AMBER94 library in the software *MOLMOL* (Koradi *et al.*, 1996 ▶). All intra-residual and inter-residual distances shorter than 5.0 Å between pairs of H atoms were then extracted and those involving labile protons with fast chemical exchange (Wüthrich, 1986 ▶) were eliminated from the resulting list. The input of upper-limit distance bounds for the structure calculation was generated by increasing these proton–proton distances by 15%. This ‘loosening’ of the distance constraints is in line with the basic strategy of interpreting ^1^H–^1^H NOEs in terms of upper-limit distance bounds (Wüthrich, 1986 ▶). For the reference NMR structure, we followed a three-step protocol: (i) a list was prepared of all the ^1^H–^1^H distances shorter than 5.0 Å in the 20 conformers that represent the NMR structure, (ii) a new list was obtained that included the longest distance among the 20 conformers for each pair of H atoms in the list resulting from (i), and (iii) the input of upper-limit distance bounds contained all entries in list (ii) that were shorter than 5.75 Å [this value was empirically selected as the shortest cutoff that gave virtually identical results for the structure calculation as an input consisting of the complete list (ii)].

### 2.7. Calculation of global displacements, global r.m.s.d.s, solvent accessibility and occluded surface packing (OSP)

The techniques used here have been described in Jaudzems *et al.* (2010 ▶). The global per-residue displacements between structure bundles refer to the mean structures calculated after superposition with minimal r.m.s.d. of the backbone-atom selections indicated in Tables 1 ▶ and 2 ▶.

## 3. Results and discussion

New NMR structures of the proteins TM1081 and A2DL1 are presented and compared with the crystal structures that have previously been determined by the JCSG. In the structure comparisons, we followed a recently introduced protocol (Jaudzems *et al.*, 2010 ▶; Mohanty *et al.*, 2010 ▶), which yielded two initial observations: (i) overall, the NMR structures of TM1081 and A2DL1 are less precisely determined than those of other proteins studied using the same protocol, as quantitated by the global r.m.s.d. values for the entire polypeptide chains, and (ii) the increased global r.m.s.d. values can be traced to discrete short polypeptide segments with high per-residue displacements. These results of the standard comparison protocol then served to guide us in devising the strategy for more detailed comparisons in §§[Sec sec3.3] [Sec sec3.4] [Sec sec3.5]3.3–3.5. Specifically, in combination with the available functional annotations of TM1081 and A2DL1, the observations (i) and (ii) revealed that residues in and near the active sites are strongly represented among the less well defined segments of the protein structures.

In order to monitor the possible impact of the different software used by the two techniques for structure calculation and refinement, we used reference crystal structures and reference NMR structures computed from the experimental structures, as described in §[Sec sec2.6]2.6 (Jaudzems *et al.*, 2010 ▶), to support the interpretation of apparent differences between the experimental NMR and crystal structures.

### 3.1. NMR structure of TM1081 and functional annotation

**Figure 1:**
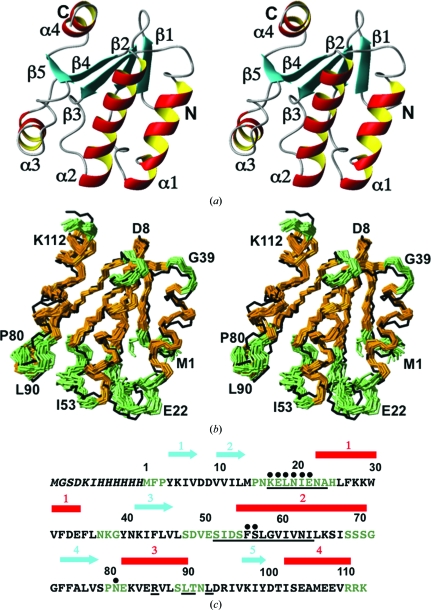
NMR structure of TM1081 and comparison with the crystal structure. (*a*) Stereo ribbon diagram of the NMR conformer closest to the mean coordinates of the bundle of conformers in (*b*). Color code: β-strands, cyan; helices, red/yellow; nonregular secondary structure, gray. The individual regular secondary structures are labeled and the N- and C-termini are indicated. (*b*) Stereoview of a superposition for best fit of the polypeptide-backbone heavy atoms of residues 3–110 of the crystal structure (black line) with the bundle of 20 conformers that represent the NMR structure. Color code used for the NMR structure: brown, residues with 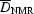 ≤ 0.50 Å, which is the mean value of the global per-residue displacements in the entire protein; green, residues with 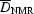 > 0.50 Å. (*c*) Amino-acid sequence of the construct used for the NMR structure determination. Black letters represent residues with 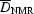 ≤ 0.50 Å and green letters those with 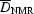 > 0.50 Å. The N-terminal segment indicated in italics originates from the expression and purification tag; it was present during the NMR measurements, but is not part of the TM1081 protein and is not shown in (*a*) and (*b*). Underlined residues were identified as being conserved in anti-σ factor antagonists (see text) in a sequence-homology search by *BLAST* and were subsequently confirmed using the *ConSurf* server (Ashkenazy *et al.*, 2010 ▶). Black dots indicate residues for which no backbone amide resonances were observed in the 2D [^15^N,^1^H]-HSQC spectrum. Above the sequence, cyan arrows indicate the positions of the β-strands and red bars those of the α-helices in the NMR structure.

The TM1081 structure contains a highly twisted five-stranded β-­sheet flanked by four α-­helices. The regular secondary-structure elements are arranged in the sequential order β1-β2-α1-β3-α2-β4-α3-β5-α4 (Fig. 1 ▶). The β-strands β2, β3, β4 and β5 (residues 11–13, 42–46, 74–78 and 98–100, respectively) are oriented parallel to each other, whereas β1 (residues 4–6) is antiparallel to β2. The α-helices α1, α2 and α3 (residues 21–34, 55–70 and 82–90, respectively) are on one side of the β-sheet and α4 (residues 104–110) is on the opposite side. Statistics of the NMR structure determination are given in Table 1 ▶ and those for the crystal structure are available from the PDB (PDB entry 3f43).

A structure-homology search using the software *DALI* (Holm *et al.*, 2008 ▶) identified ten structures with a *Z* score of ≥10. All have been annotated as anti-σ factor antagonists, share less than 25% sequence identity with TM1081 and belong to the SCOP family SpoIIaa, which includes another *T. maritima* structure determined by NMR at the JCSG, TM1442 (Etezady-Esfarjani *et al.*, 2006 ▶). The functional annotation of TM1081 is based on a sequence-homology search with *BLAST*, which showed that TM1081 contains a tripeptide Asp54-Ser55-Phe56 that forms a serine-phosphorylation motif characteristic of anti-σ factor antag­onists and also contains the following additional residues that are conserved in other anti-σ factor antagonists: Lys17–Asn23, Ser52, Ile53, Ser57–Ile64, Arg86, Leu90, Thr91 and Leu93 (Fig. 1 ▶ *c*). This analysis was confirmed by a homology search using the *ConSurf* server (Ashkenazy *et al.*, 2010 ▶) for the identification of functional regions in proteins.

### 3.2. NMR structure of A2DL1 and functional annotation

**Figure 2:**
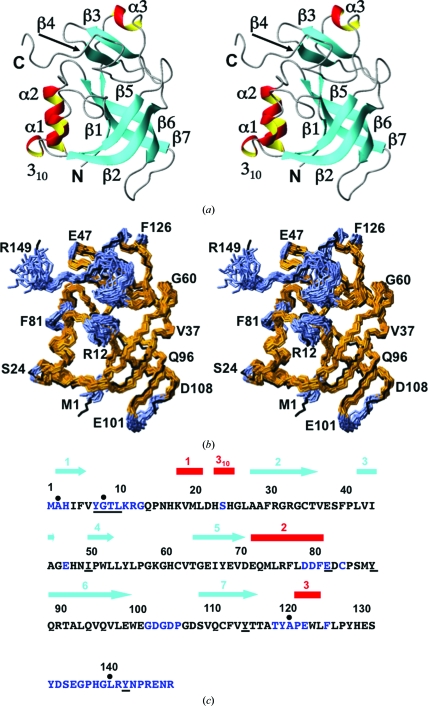
NMR structure of the protein A2LD1 and comparison with the crystal structure. The same presentation is used as in Fig. 1 ▶, but the following should be noted. In (*b*), the polypeptide-backbone heavy atoms of residues 2–100 and 106–144 were superimposed for best fit. The residues with global displacements 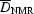 > 0.64 Å are indicated by blue coloring in (*b*) and are represented by blue letters in (*c*). In (*c*), residues forming the catalytic site are underlined (see text).

The NMR structure of mouse A2DL1 includes seven β-strands (residues 2–5, 28–36, 42–45, 50–53, 64–70, 89–99 and 109–116), three α-helices (residues 18–21, 72–81 and 122–125) and one 3_10_-helix (residues 23–25; the helical secondary-structure elements identified in the crystal structure were labeled H1–H4, with H1, H3 and H4 corresponding to α1, α2 and α3 and H2 corresponding to the 3_10_-­helix; Klock *et al.*, 2005 ▶). The sequential order of the regular secondary-structure elements is β1-α1–3_10_-β2-β3-β4-β5-α2-β6-β7-α3 (Fig. 2 ▶). The structure contains a β-barrel formed by five strands, β1-­β5-β2-β6-β7, in which strands β1 and β7 are parallel and all other neighboring strands are antiparallel (Fig. 2 ▶ *a*). The barrel is flanked on one side by helices α1 and α2, which are arranged in the direction of the barrel axis and are closest to strands β5 and β1, respectively. A two-stranded antiparallel sheet (β3–β4) is located at one end of the aforementioned β-barrel, where β4 is in contact with it. The C-terminal segment 126–149 shows no regular secondary structure and packs against sheet β3–β4. Statistics of the NMR structure determination are given in Table 2 ▶ and those for the crystal structure have been presented elsewhere (Klock *et al.*, 2005 ▶).

As described above, the functional annotation of mouse A2DL1 as a γ-glutamylamine cyclotransferase was based on comparison with the highly homologous human enzyme (Oakley *et al.*, 2010 ▶). The catalytic site of A2DL1, consisting of Tyr7, Gly8, Thr9, Leu10, Ile50, Glu82, Tyr88, Tyr115 and Tyr143 (Fig. 2 ▶ *c*), was identified based on complete conservation with respect to the human homolog. A *ConSurf* search (Ashkenazy *et al.*, 2010 ▶) for the identification of functional regions shows complete conservation for all catalytic residues, with the sole exception that, in two species, Thr9 is replaced by either Ser or Ala.

### 3.3. Global comparisons of the respective NMR and crystal structures of TM1081 and A2DL1

Following the observations described at the outset of §[Sec sec3]3, we followed a strategy of first comparing the well defined polypeptide segments with per-residue displacements below the mean values for the entire polypeptide chains, 0.50 Å for TM1081 and 0.64 Å for A2DL1, which in both proteins comprise about 65% of all residues (brown in Figs. 1 ▶ *b* and 2 ▶ *b*). Since this well defined part of the molecular structures will serve as a reference for the conclusions about the less well structured residues, we will first summarize the observations made on these scaffolds. We will then analyze the respective behavior of the less well behaved residues that are either part of the active-site regions or spatially separated from them.

**Figure 3:**
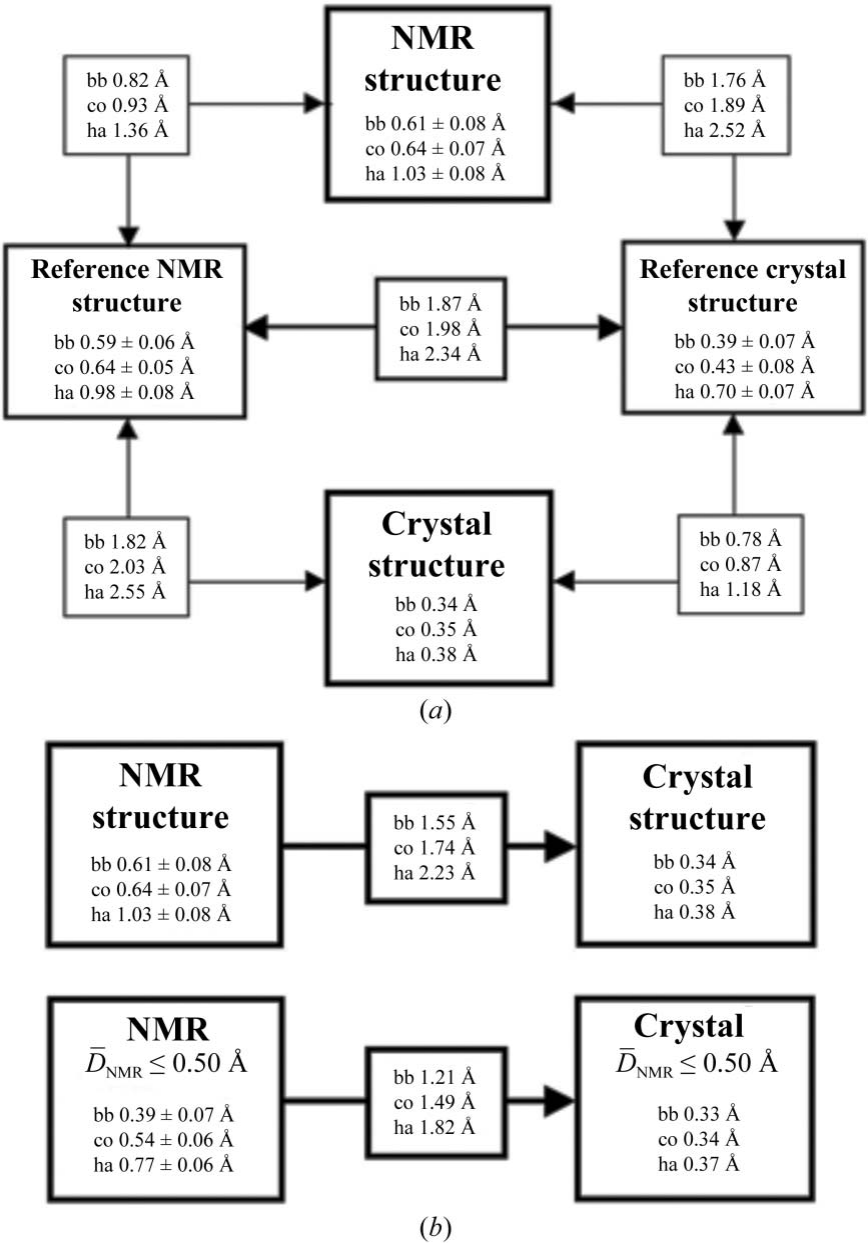
Global comparisons of the NMR structure, the crystal structure and the reference NMR and reference crystal structures of TM1081. (*a*) Global r.m.s.d. values for the NMR structure, the reference NMR structure and the reference crystal structure. The atoms used for the comparisons are bb, backbone atoms N, C^α^ and C′; co, core heavy atoms defined as having less than 15% solvent accessibility; ha, all heavy atoms. For the computation of the global r.m.s.d. values the structures were superimposed for best fit of the backbone heavy atoms of residues 3–110. For the crystal structure, we calculated an apparent global r.m.s.d. value from the per-residue displacements, which were linked to the crystallographic *B* values through an empirical scaling factor (Jaudzems *et al.*, 2010 ▶) to ensure a close match with the corresponding displacement values in the reference crystal structure (see also Figs. 9 ▶ and 13 ▶ below). For the structure comparisons, r.m.s.d. values were computed between the crystal structure coordinates and those of the conformer closest to the mean atom coordinates of each of the three ensembles of 20 conformers that represent the NMR structure and the two reference structures. Numbers framed by thick lines show the precision of the experimental structures, those with medium frames show the precision of the reference NMR and reference crystal structures and their comparison and those with thin frames show comparisons between experimental and reference structures. (*b*) Comparison of the NMR and crystal structures, with r.m.s.d.s calculated for best fit of the segment 3–110. (*c*) The same as (*b*) with r.m.s.d.s calculated for the residues with 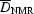 values ≤ 0.50 Å (see text).

**Figure 5:**
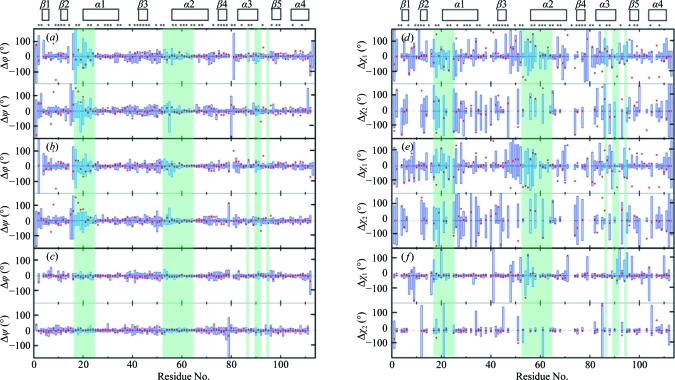
Backbone dihedral angles and side-chain torsion angles in the crystal structure of TM1081 and comparison with the NMR, reference NMR and reference crystal structures. (*a*–*c*) Spread of the values for the backbone dihedral angles ϕ and ψ in the bundles of 20 conformers representing the NMR structure (*a*), the reference NMR structure (*b*) and the reference crystal structure (*c*). In this presentation, the mean value in the bundles of 20 conformers is at 0°, the blue vertical bars represent the spread of the values within the bundles and the red dots indicate the deviation of the crystal structure values from the corresponding mean values for the bundle of 20 conformers. (*d*–*f*) Spread of the values for the amino-acid side-chain torsion angles, χ_1_ and χ_2_, in the NMR structure (*d*), the reference NMR structure (*e*) and the reference crystal structure (*f*). The same presentations are used as in (*a*)–(*c*). At the top of the two panels, the locations of the regular secondary structures are indicated and asterisks identify the residues with solvent accessibility below 15% in the NMR structure. Shading indicates the conserved residues in anti-σ factor antagonists, as in Fig. 9 ▶(*b*).

**Figure 7:**
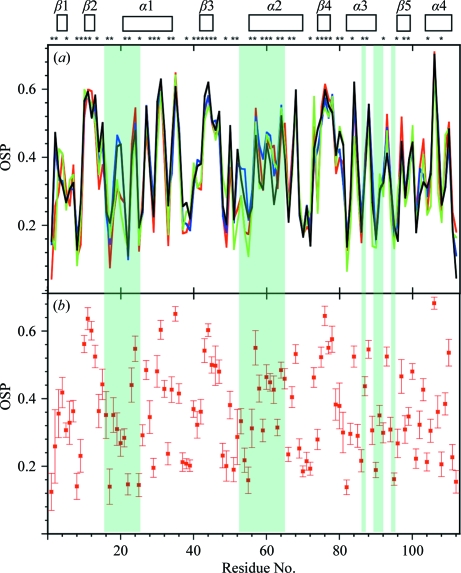
Surface packing along the polypeptide chain of TM1081. (*a*) Plots *versus* the amino-acid sequence of the per-residue occluded surface packing (OSP, a dimensionless quantity covering the range from 0.0 to 1.0; Pattabiraman *et al.*, 1995 ▶) for the NMR (red), crystal (blue), reference NMR (green) and reference crystal (black) structures. For the NMR structure and the two reference structures, the OSP values for the conformer closest to the mean atom coordinates of the bundles are shown. At the top, the locations of the regular secondary structures are indicated and asterisks identify the residues with solvent accessibility below 15% in the NMR structure. (*b*) Plot *versus* the amino-acid sequence of the mean per-residue OSP values in the NMR structure and the standard deviations among the 20 NMR conformers. Shading indentifies the conserved residues in anti-σ factor antagonists, as in Fig. 9 ▶(*b*).

For both TM1081 and A2DL1, the global r.m.s.d.s calculated for all residues with below-average displacements are similar to those reported for the previously analyzed proteins NP_247299.1 (Jaudzems *et al.*, 2010 ▶), TM1112 and TM1367 (Mohanty *et al.*, 2010 ▶) (Figs. 3 ▶ and 4 ▶). The results for the well defined protein scaffolds confirm the conclusions drawn from these earlier comparisons of NMR and crystal structures. (i) The backbone folds in the corresponding NMR and crystal structures can be overlapped with r.m.s.d. values of about 1.0 Å (Figs. 3 ▶ and 4 ▶). (ii) While the r.m.s.d. values for the backbone heavy atoms in the crystal structure are essentially identical to those for all heavy atoms, the r.m.s.d.s for the corresponding selections of atoms in the reference crystal structure differ by nearly twofold, similar to the NMR structure and the reference NMR structure (Figs. 3 ▶ and 4 ▶). (iii) Although the side-chain torsion angles show high variability in the NMR structures (Figs. 5 ▶ and 6 ▶), the packing density is closely similar to the corresponding crystal structures (Figs. 7 ▶ and 8 ▶).

Whereas very similar observations were made and near-identical quantitative results were obtained from comparison of those parts of the two proteins that are made up of residues with below-average displacements in the NMR structures, quite different insights resulted from analysis of the remaining less well structured parts of the two proteins. Therefore, the results obtained for TM1081 and A2DL1 are presented in separate sections below.

### 3.4. Analysis of the molecular regions of TM1081 with increased disorder in the NMR structure and implications for the putative functional binding site

In TM1081, the polypeptide segments with per-residue displacements above the mean value of 0.50 Å in the NMR structure consist of 39 residues, Met1–Pro3, Pro15–His25, Asn37–Gly39, Ser48–Ser55, Ser69–Gly72, Pro80–Glu82, Ser89–Asn92 and Arg111–Lys113 (green in Fig. 1 ▶ *b*), which represent 35% of the polypeptide chain. Among the 22 residues that are conserved in other anti-σ factor antagonists (Fig. 1 ▶ *c*), 14 residues, 17–24, 52–55 and 90–91, are located within these less well defined areas of the NMR structure and these will now be more closely analyzed.

**Figure 9:**
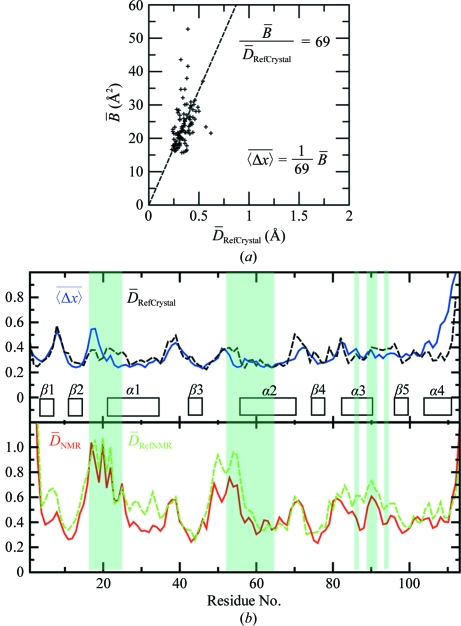
Local precision of the TM1081 structures along the sequence. (*a*) Linear least-squares fit of the crystallographic per-residue 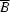 values *versus* the corresponding per-residue displacements in the reference crystal structure, 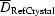, yielding* c* = 1/69 in equation (3) of Jaudzems *et al.* (2010 ▶). (*b*) Plots of the per-residue polypeptide-backbone displacements *versus* the sequence. Upper panel, crystal structure and reference crystal structure. Lower panel, NMR structure and reference NMR structure. For the crystal structure, per-residue displacements were calculated from the 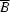 values using the relation in (*a*). For the NMR structure and the two reference structures the data correspond to the global per-residue displacements calculated for bundles of 20 conformers (Billeter *et al.*, 1989 ▶). The locations of regular secondary structures are indicated in the upper panel and conserved residues in anti-σ factor antagonists are shaded (see text).

The largest 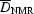 values are observed for the conserved segment Lys17–Asn23 at the start of helix α1, which is precisely structured in the crystal and also has low 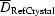 values (Fig. 9 ▶). Similarly, the large 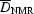 values observed for some residues in the segment 47–64, which comprises residues 47–51 that are in spatial contact with the conserved segment 17–23 and the conserved residues 52–64, contrast with their high definition in the crystal and reference crystal structures. The segment 77–95 with the conserved residues Arg86, Leu90, Thr91 and Leu93 also shows large displacements in solution that have no counterpart in the crystal structure. The low precision in segment 17–24 is also reflected in the large dihedral angle variations among the 20 conformers of the NMR structure, with six out of seven residues showing variations that exceed ±60° (Fig. 5 ▶). In the other disordered conserved segments 52–55 and 90–91, all backbone di­hedral angles are well defined in the NMR structure. In plots of the occluded surface packing (OSP; Pattabiraman *et al.*, 1995 ▶), the four experimental and reference structures display similar profiles, except that the conserved segments 17–21 and 52–58 show lower packing density in the NMR structure (Fig. 7 ▶). Overall, although the atomic coordinates of the mean NMR and crystal structures of TM1081 coincide closely throughout, increased structural disorder is manifested in the NMR data for a majority of the residues directly related to protein function (Figs. 5 ▶, 7 ▶ and 9 ▶).

It is well known that the binding of anti-σ factor antagonists is modulated by phosphorylation of a Ser residue (Ser55 in TM1081), but their mechanism of action remains elusive. Comparison of the crystal structures of the free and phosphorylated forms of the anti-σ factor antagonist SpoIIAA from *Bacillus subtilis* shows that, in contrast to other kinase-regulated protein families, phosphorylation does not seem to induce large conformational changes in the protein architecture (Seavers *et al.*, 2001 ▶). Similarly, substitution of the active Ser by an acidic residue does not mimic the effect of phosphorylation. High structure similarity was also found between the NMR structures of the free and phosphorylated forms of TM1442 (Etezady-Esfarjani *et al.*, 2006 ▶), in which the free form was extremely sensitive to variations in salt concentration and pH, while the phosphorylated form was more stable in solution. These observations have been interpreted as an indication that the role of the phosphate group is not limited to steric or electrostatic interference (Kovacs *et al.*, 1998 ▶), but also induces local structure rearrangements in the binding region (Seavers *et al.*, 2001 ▶).

**Figure 10:**
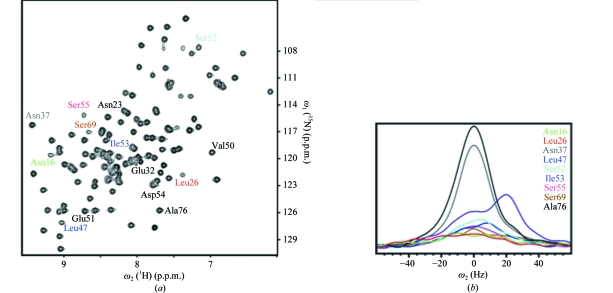
NMR evidence for slow local conformational exchange in the NMR structure of TM1081. (*a*) 2D [^15^N,^1^H]-HSQC spectrum of a 1.0 m*M* solution of uniformly ^15^N-­labeled TM1081 recorded at 800 MHz and 313 K. The cross-peaks shown in (*b*) are identified. (*b*) Cross-sections along ω_2_(^1^H) through the cross-peaks identified with the corresponding color code in (*a*).

**Figure 11:**
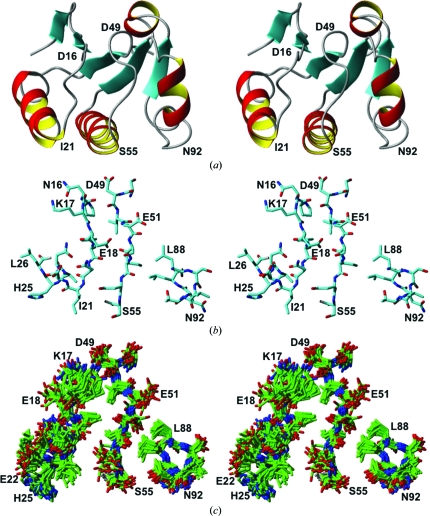
Putative active site in TM1081. (*a*) Stereo ribbon representation of the same NMR conformer as in Fig. 1 ▶(*a*) after subsequent rotations by 90° about a horizontal axis and 180° about a vertical axis. (*b*) and (*c*) show stereoviews of structural details with the same viewing angle. (*b*) Polypeptide segments 16–26, 49–55 and 88–92 in the crystal structure, which form the putative binding site of TM1081 (see text). (*c*) Bundle of 20 NMR conformers for the same segments as in (*b*).

In TM1081, the anti-σ factor binding region consists primarily of residues 17–23 and 52–55, as identified by structure homology with other anti-σ factor antagonists (Kovacs *et al.*, 1998 ▶; Etezady-Esfarjani *et al.*, 2006 ▶; Seavers *et al.*, 2001 ▶). Line broadening of amide-group signals in NMR spectra recorded at 313 K (Fig. 10 ▶) manifests con­formational exchange on the millisecond time scale for Asn16, Glu22, His25, Leu26, Phe27, Ser52–Ser55, Ser68 and Ser69. This conformational exchange involves large variations of the backbone in the segment Lys17–Ile23, which results in several charged side chains being oriented differently in solution and in the crystal (Fig. 11 ▶). In particular, whereas in the crystal structure the carboxyl group of Glu18 forms a hydrogen bond to the amide group of Ser52, it is exposed to the solvent in the NMR structure; also, the Lys17 side-chain hydrogen bond to the side-chain amide of Asn16 is not seen in the NMR structure, in which Lys17 is oriented towards Asp49. By analogy to the SpoIIAB–SpoIIAA complex, in which the crystal structure indicates that electrostatic interactions are fundamental for complex formation (Masuda *et al.*, 2004 ▶), the local rearrangement of charged residues may play an important role in modulating the affinity of TM1081 for the corresponding anti-σ factor.

Among the 25 nonconserved positions with 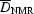 > 0.50 Å, 12 residues are located sequentially adjacent to conserved amino acids, with Pro15, Asn16 and His25 flanking the binding-site region Lys17–Ala24, segment Ser48–Glu51 preceding the conserved segment 52–64 and Ser89 and Asn92 flanking the conserved dipeptide Leu90–Thr91. In addition, segment 80–82 is spatially close to Val50 and Glu51 near the binding site. The large 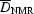 values for these residues contrast with low 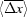 values in the crystal, similar to the observations for the conserved residues. An additional seven residues are in two solvent-exposed loops far from the binding site, *i.e.* 37–39 and 69–72, and six residues are at the chain termini. All of these residues have similar global displacements in the NMR and crystal structures.

In conclusion, in contrast to the chain termini and some solvent-exposed loops, which display expected structural disorder in solution and in the crystal, conserved binding-site segments and flanking residues that form the overall catalytic site display ‘nontrivial’, potentially function-related, disorder in the NMR structure. The solution structure and supplementary NMR data show that the binding site in the unliganded form of TM1081 undergoes slow conformational transitions on the millisecond time scale, which would allow local rearrangements triggered by functional modification of Ser55. This conformational plasticity of the unliganded form might be even more pronounced at the optimal growth temperature of 353 K for *T. maritima* and the concomitant variation of the local electrostatic charge distribution might modulate and even prevent the binding of the anti-σ factor to the nonphosphorylated form of TM1081, as previously proposed for other anti-σ factor antagonists (Kovacs *et al.*, 1998 ▶).

### 3.5. Analysis of the molecular regions of A2DL1 with increased disorder in the NMR structure and implications for the active-site conformation and functional mechanisms

**Figure 12:**
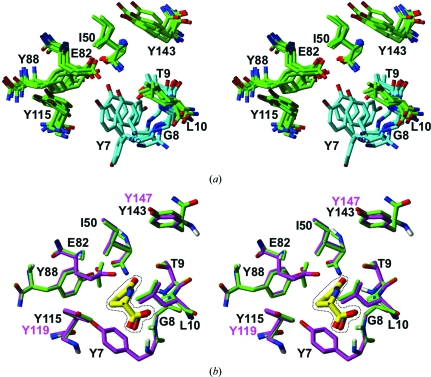
Catalytic site of the mouse γ-glutamylamine cyclotransferase A2LD1. (*a*) Catalytic site residues of A2LD1 represented by a bundle of the five lowest-energy NMR conformers. Cyan coloring highlights residues that form hydrogen bonds with the ligands in the crystal structures. (*b*) Superposition of the crystal structures of A2LD1 in complex with formate (green) and human GGACT in complex with 5-oxo-l-α-proline (magenta; PDB code 3juc; Oakley *et al.*, 2010 ▶). The two structures were superimposed for best fit of the polypeptide heavy atoms shown in the drawing, yielding an r.m.s.d. of 0.62 Å. The ligands are shown inside the dotted line as yellow carbon skeletons and otherwise with standard colors for O and N atoms. The black residue numbers are for A2LD1. Owing to an insertion, the two highest residue numbers shown in the figure for GGACT are different, as indicated in magenta.

In A2DL1, the following 46 positions have per-residue displacements 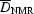 above the mean value of 0.64 Å: 1–3, 7–13, 24, 47, 79–82, 84, 102–106, 119–123, 126 and 133–149 (highlighted in blue in Figs. 2 ▶ *b* and 2 ▶ *c*). These include six of the nine catalytic site residues, *i.e.* Tyr7, Gly8, Thr9, Leu10, Glu82 and Tyr143 (Fig. 2 ▶ *c*). For these residues, we observe large per-residue NMR displacements which contrast with low *B* values in the crystal structure. Of special interest is the structural disorder of the active-site residues Tyr7, Gly8 and Thr9 in the un­liganded protein (Fig. 12 ▶ *a*), since these residues form hydrogen bonds to the substrate in the crystal structure of GGACT and to formate in the crystal structure of A2DL1 (Fig. 12 ▶ *b*). These interactions are fundamental for catalysis, as described in detail by Oakley *et al.* (2010 ▶). The three additional active-site residues, Ile50, Tyr88 and Tyr115, have high structural definition in the NMR structure, with similar side-chain orientations as in the crystal structures of A2DL1 and GGACT (Fig. 12 ▶).

**Figure 13:**
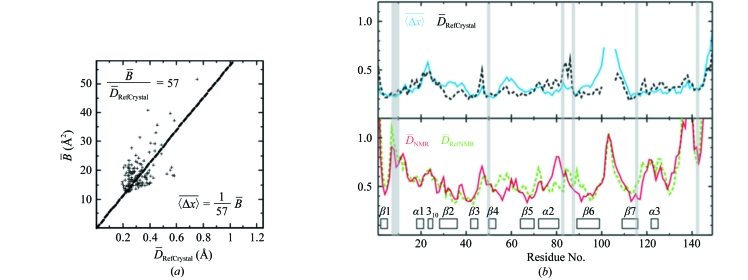
Local precision of the A2LD1 structures along the sequence. The same presentation is used as in Fig. 9 ▶. Residues of the A2LD1 active site are shaded (see text).

**Figure 14:**
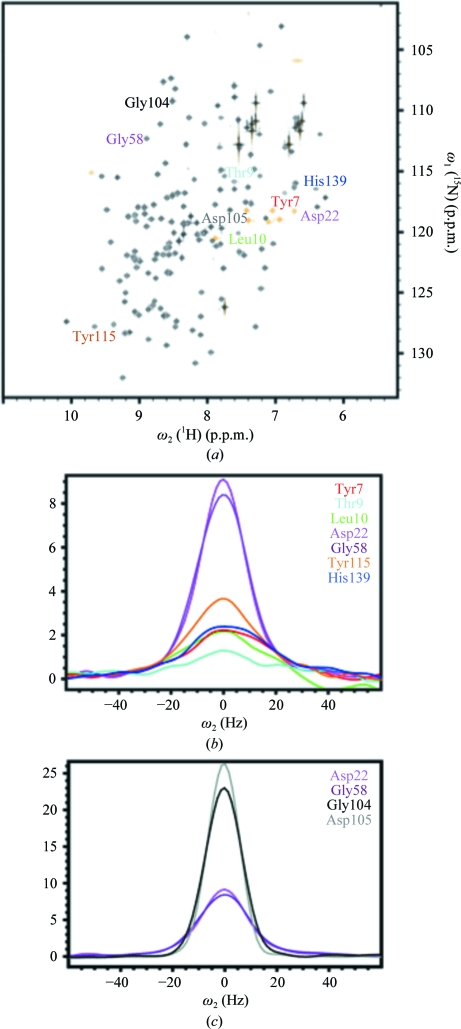
NMR evidence for local conformational exchange in the NMR structure of A2LD1. (*a*) [^15^N,^1^H]-HSQC spectrum of a 1.1 m*M* solution of A2LD1 recorded at 700 MHz and 298 K. The cross-peaks of residues shown in (*b*) and (*c*) are identified. (*b*) Cross-sections along ω_2_(^1^H) through cross-peaks of active-site residues with low-frequency mobility (see text), as identified with the corresponding color code in (*a*). (*c*) The same as (*b*) for residues with high-frequency mobility (see text).

Among the other 39 positions with 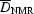 > 0.64 Å, 18 residues (11–13, 79–81, 84, 133–142 and 144) form a cavity surrounding the active site (Fig. 2 ▶ *b*), where they show similar structural characteristics as the disordered active-site residues, with high 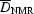 values and small crystal *B* values (Fig. 13 ▶ *b*). The common behavior of the residues in the active site and in the surrounding cavity extends to protein mobility. In the 2D [^15^N,^1^H]-HSQC spectrum of A2DL1 (Fig. 14 ▶ *a*), signals with out­standing intensities are indicated and cross-sections of these peaks are shown in Figs. 14 ▶(*b*) and 14 ▶(*c*). Variations in the relative peak intensities arise from dependence of the NMR line shapes on local intramolecular mobility. In Fig. 14 ▶, residues Asp22 and Gly58 represent line shapes that are not visibly affected by local motions. Fig. 14 ▶(*b*) shows that Tyr7, Thr9, Leu10 and Tyr115 in the catalytic cavity exhibit lower peak intensities than Asp22 and Gly58, indicating that they are subject to slow conformational exchange on the millisecond time scale. Comparable low-frequency mobility has previously been reported in many instances for residues located in catalytic sites (Wang *et al.*, 2001 ▶; Schnell *et al.*, 2004 ▶; Boehr *et al.*, 2006 ▶) and has also been correlated with ligand binding in T4 lysozyme (Mulder *et al.*, 2000 ▶).

The 21 residues with 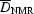 > 0.64 Å that are located outside of the catalytic cavity form the two chain termini 1–3 and 145–149 and other solvent-exposed areas far from the catalytic cavity (Figs. 2 ▶ *b* and 2 ▶ *c*). They all show poor structural definition in both NMR and crystal structures, with similar per-residue global displacement profiles (Fig. 13 ▶ *b*). For Gly104 and Asp105 in a surface loop located far from the catalytic site and not modeled in the crystal structure (Fig. 13 ▶ *b*), we observed very high peak intensities with respect to Asp22 and Gly58, and ^15^N{^1^H}-NOE measurements (data not shown) confirmed that this is a consequence of fast motion on the subnanosecond time scale.

In summary, a number of active-site and catalytic cavity residues display structural disorder in solution that is not correlated with disorder in the crystal. Supplementary NMR data further show that these residues undergo conformational exchange on the millisecond time scale, which might support controlled access of the substrate. Similar to TM1081, the conformational features of residues in and near the active site are clearly different from the ‘trivial disorder’ seen in solution, as well as in the crystals, for the polypeptide-chain termini and some peripheral surface areas distant from the active site.

## 4. Concluding remarks

There is no paucity of investigations on protein structural order/disorder and dynamics in the literature. Generally, publications in this field focus on individual targets and, for many of these proteins, an admirable wealth of detailed data has been accumulated (for recent illustrations, see Boehr *et al.*, 2010 ▶; Fraser *et al.*, 2009 ▶). In this paper, we use a protocol of systematic comparisons of corresponding structures in solution and in the crystal to investigate possible correlations of structural order and dynamics with the characteristics of the binding sites of two globular proteins. The results in the preceding sections show that the reactive areas in these two functionally annotated proteins could have been recognized from increased structural disorder and slow dynamic processes that were observed only in solution. The protein A2DL1 actually provides a striking illustration of the complementarity of relevant information collected either from the crystal or in solution; while NMR studies of the unliganded protein showed pronounced disorder for some active-site side chains, these same side chains are well ordered in the crystal structure of the ‘unliganded protein’. This different behavior can be rationalized by the observation in the crystal structure that the position of the substrate is occupied by a component of the buffer solution and that the spatial arrangement of the active-site side chains in the nonspecific complex mimics the orientation of those in the crystal structure of the homologous protein GGACT in complex with a substrate mimic. For future challenges of analyzing domains of unknown function (DUFs) with a known three-dimensional structure, the indication from the data presented here is that polypeptide segments that are differently structured in solution and in the crystal might be a first step toward obtaining function-related insights, such as identification of their active sites. Conformational exchange on the millisecond time scale for certain residues in the active site of unliganded proteins further suggests that such internal mobility, in contrast to much faster elemental thermal motions (Boehr *et al.*, 2010 ▶), must involve concerted movements of a large number of atoms with activation energies of the order of 50 kJ mol^−1^ and might be important for selective interactions with their reaction partners.

## Supplementary Material

PDB reference: TM1081, 2ka5

PDB reference: A2LD1, 2kl2

## Figures and Tables

**Figure 4:**
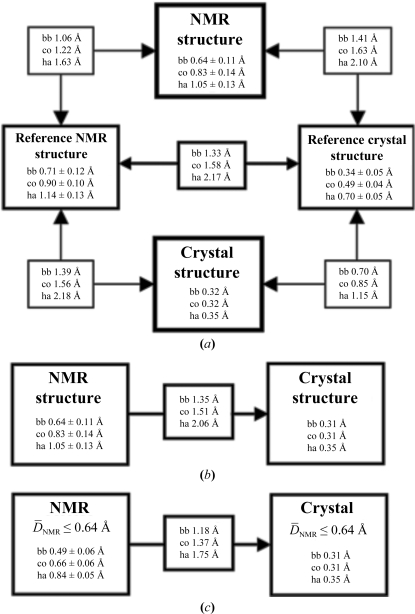
Global comparison of the crystal and NMR structures of A2LD1. The same presentation is used as in Fig. 3 ▶. For the computation of the global r.m.s.d.s the structures were superimposed for best fit of the backbone heavy atoms of residues 2–144. In (*c*), r.m.s.d.s were calculated for residues with 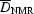 ≤ 0.64 Å (see text).

**Figure 6:**
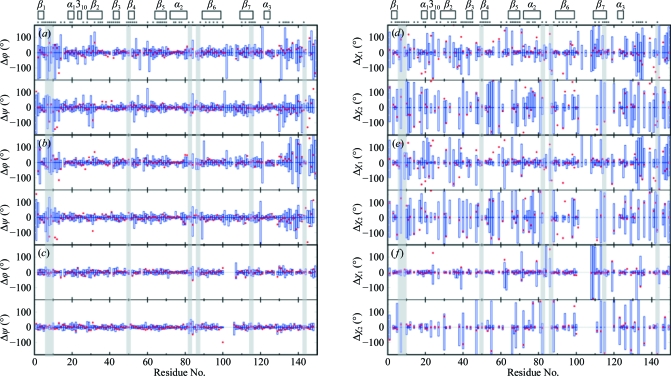
Backbone dihedral angles and side-chain torsion angles in the crystal structure of A2LD1 and comparisons with the NMR, reference NMR and reference crystal structures. The same presentation is used as in Fig. 7 ▶. Shading highlights the active-site residues, as in Fig. 13 ▶(*b*).

**Figure 8:**
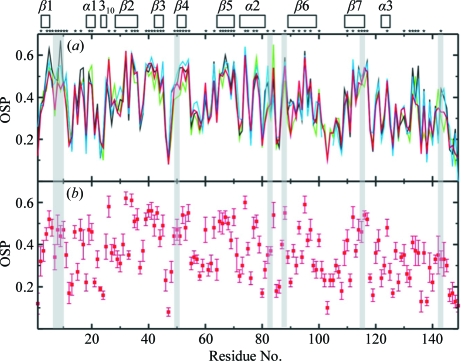
Occluded surface packing along the polypeptide chain of A2LD1. The same presentation is used as in Fig. 7 ▶. Shading identifies the active-site residues, as in Fig. 13 ▶(*b*).

**Table 1:** Determination of the NMR structure, a reference crystal structure and a reference NMR structure of the protein TM1081: input for the structure calculations and characterization of bundles of 20 energy-minimized *CYANA* conformers representing the structures Except for the top six entries and the Ramachandran plot statistics, average values and standard deviations for the 20 conformers are given.

	NMR structure†	Reference crystal structure‡	Reference NMR structure§
NOE upper distance limits	2316	4735	4055
Intra-residual	555	1035	1209
Short-range	603	1112	1075
Medium-range	554	1169	955
Long-range	604	1419	816
Dihedral angle constraints	423	413	447
Residual target-function value (Å^2^)	2.53 ± 0.29	1.17 ± 0.27	1.86 ± 0.27
Residual NOE violations			
No. ≥ 0.1 Å	47 ± 7	6 ± 2	6 ±3
Maximum (Å)	0.15 ± 0.01	0.14 ± 0.02	0.15 ± 0.06
Residual dihedral angle violations			
No. ≥ 2.5°	0 ± 0	1 ± 1	2 ± 1
Maximum (°)	2.16 ± 0.81	3.45 ± 1.09	3.86 ± 1.37
AMBER energies (kcal mol^−1^¶)			
Total	−4316 ± 121	−4323 ± 92	−4327 ± 85
van der Waals	−317 ± 15	−431 ± 18	−333 ± 11
Electrostatic	−5138 ± 107	−4720 ± 55	−4992 ± 98
R.m.s.d. from mean coordinates†† (Å)			
Backbone (3–110)	0.61 ± 0.08	0.37 ± 0.06	0.59 ± 0.07
All heavy atoms (3–110)	1.03 ± 0.08	0.71 ± 0.07	0.98 ± 0.08
Backbone ( 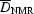 ≤ 0.50 Å)	0.39 ± 0.07	0.31 ± 0.05	0.35 ± 0.05
All heavy atoms ( 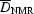 ≤ 0.50 Å)	0.77 ± 0.06	0.59 ± 0.06	0.78 ± 0.06
Ramachandran plot statistics‡‡			
Most favored regions (%)	71.4	84.8	75.3
Additional allowed regions (%)	23.8	14.7	22.6
Generously allowed regions (%)	3	0.5	2.4
Disallowed regions (%)	1.7	0.0	0.7

† Structure calculated from the experimental NMR data. The top six entries represent the input generated in the final cycle of the *ATNOS*/*CANDID* and *CYANA* calculation.

‡ Structure calculated with *CYANA* from conformational constraints derived from the molecular model representing the crystal structure and subjected to the same energy minimization as the experimental NMR structure (Jaudzems *et al.*, 2010 ▶).

§ Structure calculated with *CYANA* from conformational constraints derived from the bundle of 20 molecular models representing the NMR structure and subjected to the same energy minimization as the experimental NMR structure (Jaudzems *et al.*, 2010 ▶).

¶ 1 cal = 4.186 J.

†† The numbers in parentheses indicate the residues for which the r.m.s.d. was calculated. Residues with 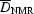 ≤ 0.50 Å are identified in Fig. 1 ▶(*c*).

‡‡ As determined by *PROCHECK* (Laskowski *et al.*, 1993 ▶). The equivalent anaysis for the crystal structure deposited in the PDB (3f34) results in 90.3% favored, 9.7% additionally allowed, 0% generously allowed and 0% disallowed.

**Table 2:** Determination of the NMR structure, a reference crystal structure and a reference NMR structure of the protein A2LD1: input for the structure calculations and characterization of bundles of 20 energy-minimized *CYANA* conformers representing the structures Except for the top six entries and the Ramachandran plot statistics, average values and standard deviations for the 20 conformers are given.

	NMR structure†	Reference crystal structure‡	Reference NMR structure§
NOE upper distance limits	3175	5557	5111
Intra-residual	615	1088	1301
Short-range	884	1415	1518
Medium-range	446	884	748
Long-range	1230	2170	1544
Dihedral angle constraints	461	502	506
Residual target-function value (Å^2^)	2.58 ± 0.31	1.86 ± 0.38	2.31 ± 0.48
Residual NOE violations			
No. ≥ 0.1 Å	28 ± 5	12 ± 2	11 ± 3
Maximum (Å)	0.15 ± 0.04	0.19 ± 0.04	0.19 ± 0.01
Residual dihedral angle violations			
No. ≥ 2.5°	0 ± 1	2 ± 1	1 ± 1
Maximum (°)	2.5 ± 1.5	3.3 ± 0.5	3.1 ± 1.4
AMBER energies (kcal mol^−1^[Table-fn tfn10])			
Total	−5427 ± 89	−5506 ± 71	−5142 ± 117
van der Waals	−512 ± 24	−464 ± 18	−377 ± 33
Electrostatic	−6276 ± 99	−6584 ± 57	−6330 ± 97
R.m.s.d. from mean coordinates[Table-fn tfn11] (Å)			
Backbone (2–144)	0.65 ± 0.11	0.34 ± 0.05	0.71 ± 0.09
All heavy atoms (2–144)	1.06 ± 0.13	0.70 ± 0.05	1.14 ± 0.09
Backbone ( 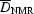 ≤ 0.64 Å)	0.49 ± 0.06	0.33 ± 0.05	0.54 ± 0.09
All heavy atoms ( 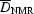 ≤ 0.64 Å)	0.84 ± 0.05	0.64 ± 0.05	0.86 ± 0.09
Ramachandran plot statistics[Table-fn tfn12]			
Most favored regions (%)	76.7	87.0	75.7
Additional allowed regions (%)	21.1	11.8	22.4
Generously allowed regions (%)	1.6	0.6	1.3
Disallowed regions (%)	0.6	0.5	0.5

† Structure calculated from the experimental NMR data. The top six entries represent the input generated in the final cycle of the *ATNOS*/*CANDID* and *CYANA* calculation.

‡ Structure calculated with *CYANA* from conformational constraints derived from the molecular model representing the crystal structure and subjected to the same energy minimization as the experimental NMR structure (Jaudzems *et al.*, 2010 ▶).

§ Structure calculated with *CYANA* from conformational constraints derived from the bundle of 20 molecular models representing the NMR structure and subjected to the same energy minimization as the experimental NMR structure (Jaudzems *et al.*, 2010 ▶).

¶ 1 cal = 4.186 J.

†† The numbers in parentheses indicate the residues for which the r.m.s.d. was calculated. Residues with 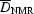 ≤ 0.64 Å are identified in Fig. 2 ▶(*c*).

‡‡ As determined by *PROCHECK* (Laskowski *et al.*, 1993 ▶). The equivalent anaysis for the crystal structure deposited in the PDB (1vkb) results in 99.3% favored, 0.7% additionally allowed, 0% generously allowed and 0% disallowed.
